# Effectiveness of the entire blood transfusion process with and without a clinical decision support system: a retrospective before-and-after study

**DOI:** 10.3389/fmed.2026.1838684

**Published:** 2026-06-22

**Authors:** Hongmei Yu, Mizhi Wu, Chen Huang, Hongying Pan

**Affiliations:** Department of Nursing, Sir Run Run Shaw Hospital, Zhejiang University School of Medicine, Hangzhou, China

**Keywords:** blood transfusion, clinical decision support system, patient safety, quality control, transfusion reaction

## Abstract

**Purpose:**

To evaluate the effectiveness of a clinical decision support system in improving management of the entire blood transfusion process using a retrospective before-and-after comparative study.

**Methods:**

A closed-loop transfusion decision support system was developed and implemented hospital-wide in 2020 following a 1-month pilot. Outcomes before implementation (Jan 2018–Jun 2020) and after implementation (Jul 2020–Dec 2022) were compared, including documentation quality and completeness, documentation time, nursing satisfaction, and transfusion adverse events.

**Results:**

After implementation, completeness of transfusion records increased from 97.1% to 99.1% (*P* < 0.05). Documentation time significantly decreased and nursing satisfaction significantly improved (both *P* < 0.001). A statistically significant difference was observed in the reported incidence of transfusion adverse reactions between the pre- and post-implementation periods (χ^2^ = 9.41, *P* < 0.05; OR = 1.39, 95% CI: 1.13–1.73).

**Conclusion:**

The system standardizes transfusion management, improves documentation completeness, reduces nursing workload, and enhances management of transfusion reactions.

## Highlights

- A whole-process blood transfusion clinical decision support system based on closed-loop management improved the standardization and traceability of transfusion practice across multiple clinical roles and workflow steps.
- Implementation of the system significantly increased the completeness of transfusion documentation, reduced nurses’ documentation time, and improved nursing satisfaction with transfusion record management.
- The system supported safer transfusion care through barcode verification, double-checking, and intelligent warning functions that strengthened nurses’ response to transfusion-related adverse reactions.

## 1 Introduction

In clinical practice, blood transfusion serves as a vital life-supporting and life-saving intervention. Whether providing support for patients with chemotherapy-induced myelosuppression or managing critical conditions resulting from severe trauma, extensive burns, and other life-threatening situations, transfusion plays an indispensable role in sustaining therapeutic progress and safeguarding patients’ survival (1–5).

Transfusion was performed in approximately 10%–15% of all hospitalizations worldwide (6). China uses more than 20 million blood component units annually, creating great challenges in transfusion safety management (7). The annual incidence rates of adverse transfusion reactions for 2018–2020 were 0.6‰, 0.7‰, and 1.0‰, with an increasing trend in incidence rates. Allergic (68.2%) and febrile non-hemolytic transfusion reactions (27.1%) were the most common (7). In high-frequency and high-risk blood transfusion therapy, any negligence or non-standard operation may affect the quality of blood transfusion and even produce serious consequences affecting patient safety. Standardized and regulated management of blood transfusion is an important guarantee of transfusion safety. In contrast, the tracking of blood products and the correct verification of information before and after blood products are the keys to guaranteeing blood transfusion safety.

The entire blood transfusion process involves three roles blood bank, clinician, and clinical nurse, and 7 necessary links from storage to blood bag recovery, which requires scientific, reasonable, and perfect management of the entire blood transfusion process to ensure the safety of clinical blood transfusion. Closed-loop management is the entire management process as a closed-loop system, forming a “decision-making, control, feedback” cycle, and continuous improvement and refinement (8). Our hospital has implemented blood transfusion information management since 2018. We found some problems in blood transfusion care and quality management during the system application process. In the current common closed-loop practice of blood transfusion, the blood department must be notified by phone after the blood bank has finished preparing blood, and there is the risk of verbal communication and lagging problems. Nursing blood transfusion documentation is complex, easy to record untimely, inaccurate, and inconsistent medical and nursing records and other issues, to medical disputes buried hidden dangers.

The new electronic system is an inevitable outcome of the standardization of nursing information, and it is also the most convenient and effective tool in the nursing field (9, 10). It can greatly improve the work efficiency of nurses, effectively reduce errors, and support clinical decision-making (11, 12). Clinical Decision Support System (CDSS) is a computer system that supports clinical decision-making, utilizes computers to use data fully, and improves and enhances decision-making efficiency through human-computer interaction (13, 14). The hospital established a special project team from May 2020 to July 2020 to build a Whole-process blood transfusion closed-loop clinical decision support system and conduct it throughout the hospital. Improving the electronic structure of blood transfusion medical records is setting up blood transfusion-related nursing warnings and nursing decisions, and realizing automatic data flow, supervision, monitoring, analysis, and evaluation. During a blood transfusion, further improving blood use standards, the final goal is to ensure the clinical use of patients.

### 1.1 Aim

The purpose of this study is to validate the effectiveness and clinical applicability of our in-house developed clinical decision support system for the entire blood transfusion process. This study proposed the following two research hypotheses:

H1: Compared with the pre-implementation period, implementation of the whole-process blood transfusion clinical decision support system would improve nurse-related transfusion management outcomes, including increasing the completeness of transfusion records, reducing the time required for nurses to complete transfusion documentation, and improving nurses’ satisfaction with transfusion documentation.

H2: Compared with the pre-implementation period, implementation of the whole-process blood transfusion clinical decision support system would reduce the incidence of transfusion adverse events or transfusion-related accidents, thereby improving the safety of clinical blood transfusion management.

## 2 Materials and methods

### 2.1 Construction of blood transfusion clinical decision support system

#### 2.1.1 Formation of the specialized transfusion project team

A blood transfusion management group was established, consisting of 7 members. The team included the Deputy Director of Nursing (responsible for nursing information), the Head Nurse of the Hematology Department, a doctor, specialized nurses in information technology, staff from the hospital’s IT department, personnel from the transfusion department, and staff from the hospital’s quality management department (responsible for transfusion quality control inspections). The Deputy Director of Nursing (responsible for nursing information) served as the team leader, the Head Nurse of the Hematology Department coordinated organizational efforts in each nursing unit, specialized nurses in information technology coordinated between the Nursing Department and the Hospital Information Coordination Group, IT department personnel were responsible for system corrections and improvements, transfusion department personnel provided professional advice on transfusion-related knowledge and quality management department staff was responsible for checking and giving feedback on transfusion quality control data.

#### 2.1.2 System design and hardware configuration

Taking closed-loop management as the theoretical basis, we develop a whole-process blood transfusion clinical decision support system based on clinical Blood Transfusion Guidelines, Blood Transfusion Medical Record Writing Quality Evaluation Criteria Sheet, and other related literature (15, 16). The system functions include blood bank management, physicians applying for blood, and nurses executing blood use, based on the division of roles and platform permissions, matching the corresponding server architecture and client. The whole-process blood transfusion clinical decision support system supports the cooperation of a computer (PC) and a palmtop computer (PDA). At the same time, we have designed the blood transfusion medical advice, patient’s informed consent for blood transfusion, electronic blood application form, blood application list, patient’s blood type related information, blood preparation related information, blood collection, mobile scanning blood collection and blood transfusion module, and realized two-person verification in the blood transfusion module, which provides the corresponding functional support for the whole-process blood transfusion clinical decision support system.

#### 2.1.3 Establishment of the knowledge base for the transfusion CDSS

The project development team conducted literature reviews on transfusion-related topics, covering work processes, record requirements, scoring rules, human-computer interaction, patient symptoms, signs, diagnosis and treatment related to transfusion adverse reactions, and educational rules. The project team organized expert discussions on the knowledge base related to the transfusion nursing decision support system and conducted research and analysis on transfusion-related quality indicators, gradually refining the knowledge base. The rules of the transfusion-related knowledge base include time limits, numerical limits, qualification and eligibility, document integrity, information consistency, logical sequence, and other aspects. The system ensures controlled processes for transfusion nursing by associating with the backend knowledge base. Examples of the rule categories include ① Time limits: setting permissions for modifying electronic medical records for transfusion within specific shifts, with modifications allowed only by the individual and the head nurse within the patient’s hospitalization period. The record cannot be modified after the patient is discharged. According to institutional transfusion practice regulations in China, blood transfusion is generally required to be completed within 4 h after blood issuance; however, this requirement was managed clinically rather than automatically enforced by the system backend. ② Numerical limits: setting upper and lower limits for vital signs in the backend, triggering warning alerts if exceeded. Error messages are linked to transfusion-related vital sign numerical inputs, preventing saving if the input is outside specified limits. For instance, if the upper limit of temperature is set below 35 degrees Celsius or the lower limit above 43 degrees Celsius, an error message will appear for blood pressure input format errors (lacking “/”), non-numeric format for numerical attribute vocabulary input, and non-date format for date attribute vocabulary input. ③ Qualification and eligibility: setting qualifications and operational permissions for nursing personnel authorized to perform transfusions, standardizing nursing processes. ④ Document integrity: setting mandatory terms in the structured electronic transfusion medical record vocabulary to ensure document completeness. ⑤ Information consistency: using the patient’s unique medical record number for barcode scanning to confirm patient information. Double confirmation is required for matching patient information such as blood type, allergy history, orders, medication contraindications, etc. ⑥ Logical sequence: automatically linking the writing order of transfusion electronic medical records in the backend and prompting the correct sequencing of nursing records according to regulations.

#### 2.1.4 Environment construction for system development

The development environment of the Transfusion CDSS is based on B/S (Browser/Server) technology, Microsoft. NET Framework 4.5 development environment, and SQL Server database server. It is designed and developed for computer use in the Windows operating system environment (both 32-bit and 64-bit). The system is designed to retrieve data views from the Hospital Information System (HIS). The client-side, utilizing PDA, is developed using JAVA language, integrating mobile computing, wireless LAN technology, and middleware technology to retrieve data views from the computer application system: Barcode and RFID technology link transfusion orders and related project information during transfusion execution.

### 2.2 Application of entire blood transfusion process

#### 2.2.1 Participants and procedures

The blood transfusion system was completed in June 2020 and underwent clinical testing in the general surgery department. The trial lasted for 1 month and included a total of 100 patients. Due to incompatibility between specialized departments (intensive care unit, operating room, emergency department) and inpatient nursing workstations, 120 nurses from inpatient ward were selected using a random number table method to document transfusion record time consumption and evaluate system satisfaction before and after implementation.

**FIGURE 1:**
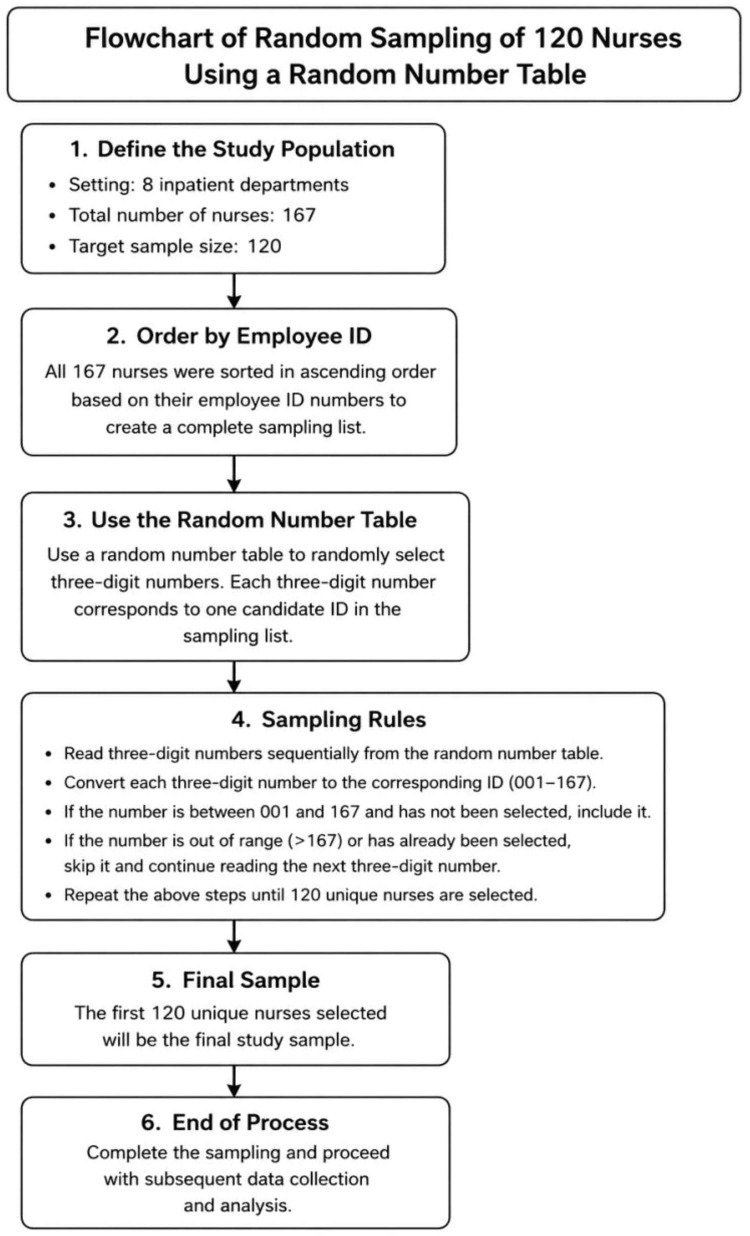
Selection of nurses.

Inclusion criteria for nurses were: registered nurses working in inpatient ward; employment duration exceeding 6 months prior to system implementation; voluntary participation. Withdrawal criteria specified that: participants could withdraw from the study at any time. The nurse selection process is shown in Figure 1.

#### 2.2.2 Measures and data collection

A comparative analysis was conducted between the post-implementation period (July 2020 to December 2022) and the pre-implementation period (January 2018 to June 2020) to evaluate hospital-wide quality of transfusion nursing documentation and completeness of transfusion protocols. Documentation quality was defined as the degree to which transfusion nursing records met institutional requirements for timeliness, accuracy, completeness, logical consistency, standardized terminology, and conformity with transfusion documentation procedures. The quality of transfusion nursing documentation was assessed using the institution’s established Checklist for Quality Assessment of Blood Transfusion Documentation (see Table 1). This instrument, with a maximum score of 100 points, assigns specific weights to individual items. Evaluation was performed through a cross-departmental peer-review process, whereby head nurses from non-native departments assessed the documentation quality of participating units.

**TABLE 1:** Checklist for quality assessment of blood transfusion documentation.

Content		Score
Dual signatures on transfusion report		3
Dual signatures for medication order execution		5
Transfusion orders are executed via the EDA		5
Blood products must be transfused within 30 min of dispatch from the blood bank		5
Pre-transfusion documentation (to be completed within 1 h)	Time	1
	Vital signs	4
	Type of vascular access	2
	Condition of the infusion site	2
	Pre-transfusion medication administration	2
	Education	2
Initiation of transfusion	Time	2
	Vital signs	2
	Vascular access	2
	Flow rate	2
	Condition of the infusion site	2
	Blood type	2
	Transfusion volume	2
	Blood components	1
	Blood bag ID	1
	Reflection	2
	Education	2
Record at 15 min	Vital signs	4
	Vascular access	2
	Flow rate	2
	Condition of the infusion site	2
	Reflection	2
	Timeliness	2
Hourly recording	Vital signs	2
	Vascular access	2
	Flow rate	2
	Condition of the infusion site	2
	Reflection	2
	Timeliness	2
Transfusion completion record	Vital signs	4
	Vascular access	2
	Condition of the infusion site	2
	Reflection	2
Record at 1 h post-transfusion	Vital signs	4
	Reflection	2
Document in accordance with protocols		1
Documentation within the closed loop		1
Total infusion time complies with policy		5
Total		100

For documentation record sampling, one transfusion documentation record filed under each nurse’s unique identifier was retrieved from the electronic medical record system for both the pre-implementation and post-implementation periods. If multiple transfusion records were available for the same nurse within either period, one record involving Packed Red Blood Cells PRBCs or plasma transfusion was randomly selected using a random number table method. This approach was used to ensure that each nurse contributed one comparable documentation record before and after implementation.

Documentation time was measured as the time required to complete electronic transfusion documentation on the computer, from opening/initiation of the transfusion documentation interface to final submission or saving of the completed record. The documentation time of the selected pre-implementation record was compared with that of the selected post-implementation record for each nurse.

The completeness of transfusion documentation was similarly reviewed by head nurses from other departments. This assessment focused on the integrity of transfusion medical record entries–specifically, the absence of missing, omitted, or incomplete items–to derive a completeness score.

Among the randomly selected 120 nurses, one transfusion documentation record filed under each nurse’s unique identifier was retrieved from the electronic medical record system for both pre-June 2020 and post-June 2020 periods. When multiple transfusion records existed for a nurse, one record documenting either red blood cell or plasma infusion was randomly selected using a random number table method. The time required for electronic documentation–from initiation to completion on the computer–was compared between the two records.

Satisfaction surveys were administered to the same 120 nurses before and after system implementation, utilizing a self-designed 5-point Likert-scale item to assess satisfaction with the electronic transfusion documentation (where 5 = very satisfied, 4 = satisfied, 3 = neutral, 2 = dissatisfied, and 1 = very dissatisfied).

Through the hospital quality office, the quality management system was used to aggregate the total number of transfusion cases and incidents of transfusion adverse events or accidents–including acute intravascular hemolysis, delayed adverse transfusion reactions, anaphylaxis, non-hemolytic febrile reactions, urticaria, transfusion-transmitted infections, circulatory overload, and blood contamination–for January 2018 to June 2020 and July 2020 to December 2020. Overall differences between these periods were compared.

#### 2.2.3 Data analysis

Descriptive statistics were applied to characterize the baseline demographics of the finally included nurses. Statistical analyses were performed using SPSS version 27.0. Paired-sample *t*-tests were employed to compare Documentation Quality, Documentation Completeness, Documentation Time, and Satisfaction with the System. The difference in the incidence of transfusion adverse events between the pre- and post-implementation periods was assessed using the Chi-square test. The Odds Ratio (OR) with its 95% confidence interval was calculated directly from the 2 × 2 contingency table.

### 2.3 Ethics approval

This study received approval from the Ethics Committee of ******* (Approval No. 20241058). This study was designed as a retrospective observational study based on existing institutional clinical and quality management data. Ethical approval was obtained in August 2024 prior to retrospective data extraction and analysis.

## 3 Results

### 3.1 Results of the entire blood transfusion process clinical decision support system

#### 3.1.1 Design of the entire blood transfusion process clinical decision support system

① Blood application

The starting point is that the doctor writes a blood transfusion order in the hospital information system (HIS), the system automatically brings in the basic information of the electronic application form, the blood type is imported from the original blood type, and the blood type applied for must be consistent with the original blood type. If it is inconsistent, the system will give a prompt and the subsequent process cannot be carried out.

② Collection of blood specimens

The system will automatically generate a “crossmatch” barcode based on the electronic blood request form, i.e., when requesting PRBCs or whole blood, a crossmatch test barcode will be automatically generated in the blood preparation field of the nurse information system. If the patient does not have a blood collection task, the system will indicate no need to prepare blood. If there is a blood collection task, the task will appear in the pending interface, and then scan the barcode label, the two match, that is, start collecting specimens, specimen collection is complete, the task automatically disappears from the pending interface, and at the same time, record the start of the collection, the end of the collection of time and collection of the nurse’s name. The nurse records the time of specimen delivery by scanning the test barcode, and the blood bank staff scans the barcode to complete the handover. According to institutional transfusion practice regulations, pre-transfusion blood specimens remained valid for 72 h. The system automatically verified specimen validity based on collection time and generated reminders when repeat specimen collection was required.

③ Blood preparation

After the blood bank receives the specimen and prepares blood according to the blood application form, if the blood type of the prepared blood is the same as the patient’s blood type, the type of the prepared blood is the same as the applied type. If the quantity of the prepared blood is ≤ the amount applied, the blood preparation will be completed. Otherwise, the system will pop a reminder box and terminate the process.

④ Sending and taking blood

Blood bank personnel to prepare blood, such as the application for blood type and the type of blood preparation is not consistent with the clinician can be used after communication, you must enter the reason, such as doctors apply for “PRBCs,” but the blood bank blood preparation for “de-albuminized PRBCs,” the system will be a pop-up box to remind. It must be entered into the non-space reason. The blood bank staff prepares the blood and asks the nurse over the phone if they can send the blood. After the blood bank personnel checks that the blood type is correct, go to the blood bank to collect blood and click on the two-dimensional code of the electronic blood collection form when collecting blood. After scanning the two-dimensional code of the blood collection form, the blood bank staff will scan the exclusive two-dimensional code including the nurse’s name and work number to complete the blood distribution step. The nurse will carry the blood to the ward after checking that the information on the blood bag is correct with the patient’s information.

⑤ Blood check by nurse

After checking the quality of the blood, the nurse scans the two-dimensional code on the blood bag, and the nurse’s workstation terminates with the patient’s basic information, such as name, bed number, hospitalization number, blood bag number, blood type, cross-matching results, blood type, and blood dosage. Two nurses check the blood against the patient’s information, and if there is a match, the second nurse’s job number and password are entered to complete the blood collection process.

⑥ Blood transfusion

Scanning the patient’s wristband and blood label in the PDA blood transfusion module shows successful execution, and the 2nd nurse scans again to complete the double check.

⑦ Blood bag recycling

Through the blood bag recovery module to complete, the system also records the blood bag recovery nurses, laborers, and blood bank personnel’s names and the time of each node, to complete the whole process of blood transfusion. Through the blood bag recovery module, the system also records the names of blood bag recovery nurses, sending workers and blood bank personnel, and the time of each node, thus completing the entire blood transfusion process.

#### 3.1.2 Design of the blood transfusion nursing intelligent warning system

**FIGURE 2:**
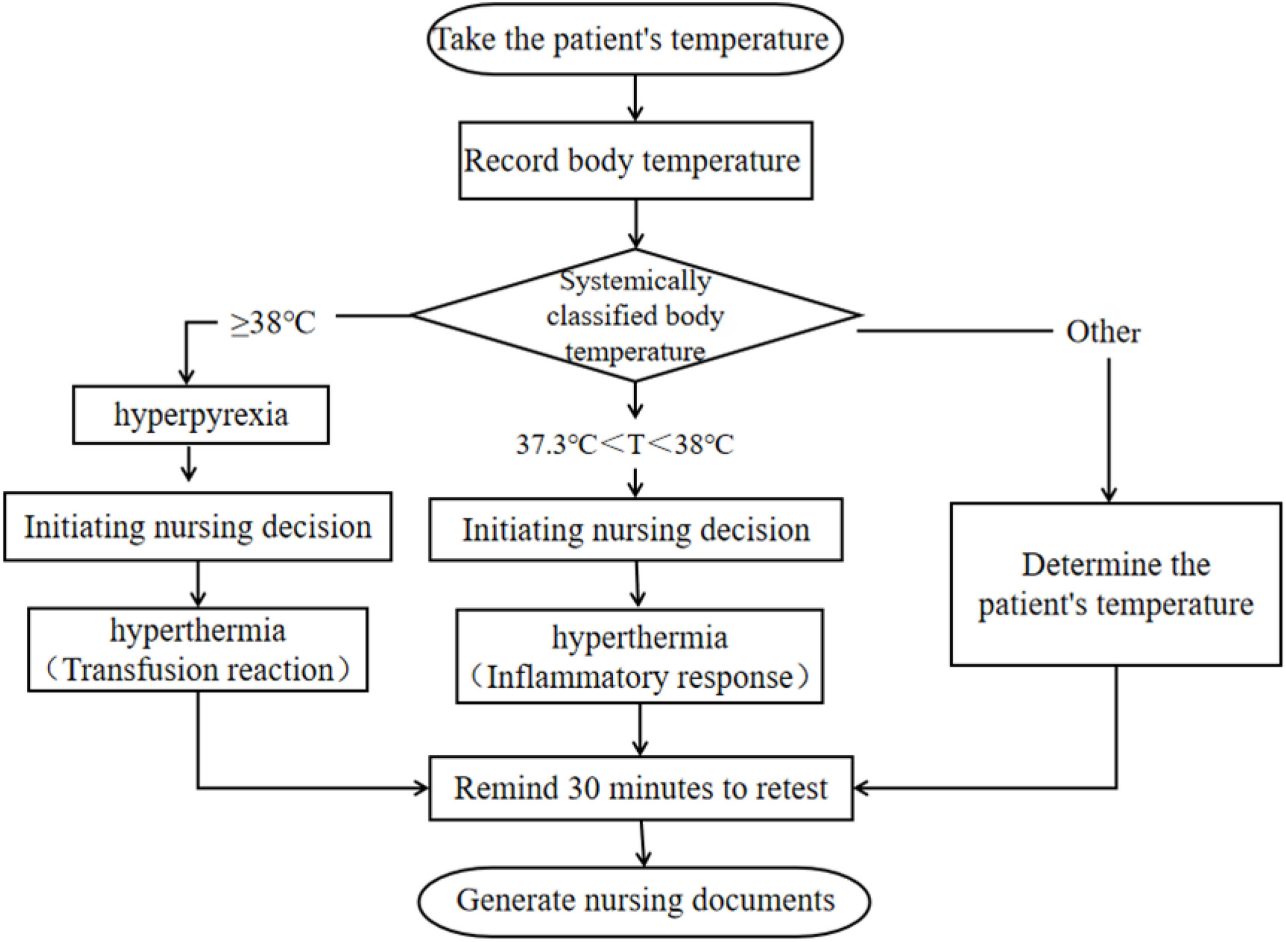
Nursing alert decision related to body temperature.

The transfusion nursing intelligent warning system triggers information reminders and warnings based on the record content of the structured electronic transfusion medical record, vital sign values, and the sequence before and after input. When entering sign values related to transfusion, the backend sets upper and lower limits to trigger warnings. The alarm decision uses an example of high body temperature (see Figure 2). If the patient’s temperature exceeds 37.3 °C or 38 °C, entering the temperature triggers a warning automatically. Intelligent nursing decisions related to temperature are presenting, displaying patient nursing diagnosis, nursing goals, nursing measures, and nursing education content. After nurse confirmation, nursing orders generated automatically. The content of temperature-related nursing warning decisions is shown in Figure 1. The design of the transfusion nursing intelligent warning system helps nurses carry out more standardized responses to transfusion-related reactions.

### 3.2 Results of application of entire blood transfusion process

#### 3.2.1 Basic information of nurses participating in system implementation testing

Among the randomly selected 120 nurses, one ultimately declined to participate in the post-implementation survey, resulting in a final dataset of 119 participants. The cohort consisted exclusively of female nurses, with the majority concentrated in the 20–29 age range and relatively junior in terms of work experience. Distributions of Marital Status, Nurse Hierarchy, and Nursing Professional Ranks were generally balanced across the sample. Detailed characteristics are presented in Table 2.

**TABLE 2:** Basic information of nurses participating in system implementation testing.

Category	Subcategory	Number
Gender	Men	0
	Women	119
Age	20–29	72
	30–39	37
	40–49	9
	50–59	1
Marital status	Single	68
	Married	51
Nurse hierarchy	Grade1	21
	Grade2	23
	Grade3	30
	Grade4	45
Nursing professional ranks	Registered nurse	23
	Senior nurse	51
	Nurse-in-charge	40
	Associate professor of nursing	5
Years of experience (year)	1–5	73
	6–10	16
	11–15	13
	16–20	8
	21–25	4
	26–30	4
	31–35	1

#### 3.2.2 Transfusion nursing medical record quality

A comparative analysis of hospital-wide transfusion documentation quality control scores, organized by the Nursing Department between January 2018-June 2020 and July 2020-December 2022, revealed no statistically significant improvement in overall documentation quality following implementation of the full-process transfusion clinical decision support system (*P* > 0.05). However, the completeness of transfusion documentation demonstrated a statistically significant increase from 97.1% to 99.1% (*P* < 0.05).

#### 3.2.3 Comparison of documentation time and satisfaction with the system

Following system implementation, the time required for clinical nurses to perform transfusion operations and complete documentation was significantly reduced compared to the pre-implementation period, with a statistically significant difference (*t* = 35.78, *P* < 0.001). Furthermore, nurses’ satisfaction with transfusion documentation showed a statistically significant improvement after system implementation (*t* = −44.14, *P* < 0.001). Detailed results of the paired-sample *t*-tests are presented in Table 3.

**TABLE 3:** Comparison of time consumption and job satisfaction before and after implementation of the blood transfusion CDSS.

Outcome measure	Mean difference	Std. deviation	Std. error mean	95% confidence interval of the difference		t	df	Sig. (2-tailed)
				Lower	Upper			
Documentation time	2.87	0.88	0.08	2.71	3.03	35.78	118	<0.001
Satisfaction with the system	−1.97	0.49	0.05	−2.06	−1.88	−44.14	118	<0.001

Std, standard deviation; df, degrees of freedom; Sig, significance.

#### 3.2.4 Incidence of transfusion adverse events or transfusion-related accidents

During the 6-months period preceding system implementation, a total of 11198 transfusion cases were recorded hospital-wide, with 131 adverse transfusion reactions reported. These included 2 cases of acute intravascular hemolysis, 1 case of anaphylaxis, 52 cases of non-hemolytic febrile reactions, 75 cases of urticaria, and 1 case of blood contamination.

In the 6 months following system implementation, 16582 transfusion cases were recorded, during which 268 adverse transfusion reactions were documented. These consisted of 2 cases of anaphylaxis, 6 cases of delayed adverse transfusion reactions, 153 cases of non-hemolytic febrile reactions, 98 cases of urticaria, 1 case of transfusion-transmitted infection, 7 cases of circulatory overload, and 1 case of blood contamination.

A statistically significant difference in the incidence of reported transfusion adverse reactions was observed between the pre- and post-implementation periods (χ^2^ = 9.41, *P* < 0.05). The Odds of reported transfusion adverse reactions were higher in the post-implementation period than in the pre-implementation period (OR = 1.39, 95% CI: 1.13–1.73). Notably, no cases of acute intravascular hemolysis were reported after system implementation. Detailed data are presented in Table 4.

**TABLE 4:** Comparison of incidence of transfusion adverse events or transfusion-related accidents before and after implementation of the blood transfusion CDSS.

Statistical test	Value	df	Asymptotic significance (2-sided)	Exact Sig. (2-sided)	Exact Sig. (1-sided)
Pearson chi-square	9.41^a^	1	0.002	0.002	0.001
Continuity correction^b^	9.09	1	0.003		
Likelihood ratio	9.64	1	0.002		
Fisher’s exact test					
Linear-by-linear association	9.41	1	0.002		
N of valid cases	27780				

^a^0 cells (0.0%) have expected count less than 5. The minimum expected count is 160.84.

*^b^*Computed only for a 2 × 2 table. df, degrees of freedom; Sig, significance.

## 4 Discussion

Blood transfusion organizations should establish a comprehensive quality management system encompassing all aspects of production and service, capable of continuous improvement, emphasizing controlling the entire blood-to-vessel transfusion chain (17). The control of the entire blood transfusion process chain should be noted. Clinical blood transfusion quality management is a crucial component of hospital quality management (18). CDSS logically controls the closed loop of the whole process of blood transfusion, and each link must be executed step by step according to the process, without any possibility of omission or jumping, thus ensuring the standardized operation of all the links.

A recent UK survey encompassing 114 hospitals (19) revealed that only 14.2% of hospital information systems contained a transfusion clinical decision support module–a key indicator of advanced healthcare informatization. In contrast, as a developing country, China has witnessed the development of a closed-loop transfusion management system at our institution that fully leverages the advantages of CDSS to achieve comprehensive system coverage across all scenarios and the entire transfusion process. Regardless of the clinical setting or underlying etiology necessitating transfusion, this system provides full-process intelligent assistance for each transfusion event, representing a breakthrough achievement for Chinese healthcare institutions in transfusion safety management and health informatization development.

Before constructing the Blood Transfusion CDSS, the development project team established the system’s knowledge base through multiple meetings and discussions by researching and analyzing quality indicators in the closed-loop blood transfusion process. The knowledge base includes time limits, numerical limits, qualifications and capabilities, document integrity, information consistency, logical order, etc. The Blood Transfusion CDSS ensures process control by associating with the background knowledge base. Through information feedback control via information technology, the system provides the correctness and completeness of the transfusion nursing process data. It guides clinical nurses to conduct nursing work homogeneously through intelligent decision-making based on data. Subsequently, data is re-extracted to generate transfusion medical record quality control forms, improving the efficiency of clinical nurses and enhancing the efficiency of quality control in the nursing management department. A comparison of transfusion documentation entry completeness before and after implementation - specifically regarding the presence of missing or incomplete items - demonstrated significant improvement in completeness rates. This enhancement is attributable to the incorporation of information integrity control requirements within the quality control process design.

In recording structured electronic nursing records for transfusion, the Transfusion Nursing Decision Support System triggers prompts through recording, targeting specific numerical values, missing or incomplete items, errors, or unwritten items set in the background to be unsavable. Following system implementation, the time required for clinical nurses to perform transfusion procedures and complete documentation was significantly reduced compared to the pre-implementation period, with a statistically significant difference (*P* < 0.05). This finding demonstrates the effectiveness of the Clinical Decision Support System (CDSS) in enhancing clinical efficiency, which aligns with previous research (20–22).

Through the closed-loop management construction of information, based on the principle of interconnection and interoperability, the blood transfusion closed-loop system constructed based on CDSS realizes the information sharing among doctors, nurses, and technicians, improves the working efficiency of the three parties, and guarantees the safety of clinical blood (23). Clinical staff also subjectively believe that the system can reduce the errors of medical staff in the process of blood transfusion, improve the safety of blood, improve the efficiency of medical staff, standardize the staff’s work behavior, and simultaneously meet the clinical needs. Therefore, the satisfaction scores of the blood transfusion system for nurses in this study were all greater than 4 points (total score of 5 points).

The present study observed a higher reported incidence of transfusion adverse reactions following implementation of the CDSS. This finding should be interpreted cautiously. Rather than indicating a deterioration in transfusion safety, the increase in reported reactions may reflect enhanced surveillance sensitivity, improved documentation completeness, and increased reporting awareness associated with the CDSS and its integrated warning functions. The system established standardized monitoring and structured recording processes for transfusion-related vital signs and adverse reactions, which may have facilitated earlier identification and more consistent reporting of transfusion-related events. In particular, the intelligent warning functions and mandatory documentation requirements may have reduced underreporting of mild or transient transfusion reactions that might previously have been overlooked in routine clinical practice. At the same time, no cases of acute intravascular hemolysis were reported after system implementation. Although causal inference cannot be established in this retrospective study, this observation may suggest that the barcode verification, double-check procedures, and information consistency control embedded within the CDSS contributed to safer transfusion practices. Because the current study design cannot distinguish between a true increase in adverse reactions and improved event detection/reporting, the adverse event findings should primarily be interpreted as reflecting enhanced hemovigilance and standardized transfusion monitoring following CDSS implementation.

This enhanced hemovigilance is technically supported by the system’s early warning mechanisms established for specific physiological thresholds. After entering the patient’s transfusion-related information into the system, the system can identify the patient’s early condition based on parameters such as respiration, blood pressure, and temperature, providing a prompt warning. The warning prompts medical staff to take corresponding nursing measures. In practice, due to differences in the seniority or ability of nurses, there are differences in their clinical decision-making abilities (24). The warning system can alert transfusion-related risks, reducing errors in nursing decision-making due to subjectivity.

The presence of the early warning system may also improve nurses’ reporting rates for adverse events, which we believe explains the observed increase in reported transfusion reactions following system implementation. However, this interpretation requires further empirical validation through dedicated studies. When nurses select transfusion adverse reactions, the system prompts corresponding nursing measures, effectively improving the correctness and capabilities of nurses in handling adverse transfusion reactions, further ensuring patient safety. Nevertheless, the present retrospective study is subject to certain limitations regarding data granularity and process-level metrics. Due to the constraints of the institutional database, detailed process-level safety indicators–such as two-person verification adherence, precise transfusion timing metrics, and blood wastage–were not systematically archived for analysis. Future prospective studies could address these gaps by incorporating broader hemovigilance indicators (such as near-miss events and specimen identification errors), quantitative process KPIs, and internationally recognized transfusion safety frameworks to provide a more comprehensive evaluation of closed-loop, CDSS-supported transfusion management.

### 4.1 Limitations

First, the retrospective before-and-after design is susceptible to temporal confounding. Secular trends during the study period–such as evolving hospital workflows amid COVID-19 and growing staff familiarity with transfusion protocols–might partially contribute to the improved outcomes, meaning the effects cannot be isolated solely to the CDSS. Second, while the study-specific evaluation tools lack formal prior validation, their standardized criteria and long-term institutional use by systematically trained evaluators ensured data reliability. Finally, limited by the lack of continuous, time-point-specific data in our retrospective dataset, we utilized aggregate-level assessment rather than an interrupted time series analysis. In addition, the exclusion of high-acuity transfusion settings, such as intensive care units, emergency departments, and operating rooms, may limit the generalizability of the findings. Future prospective or multi-timepoint studies are warranted to better control for these contextual factors and to evaluate CDSS implementation in high-risk transfusion settings using setting-specific workflow integration approaches.

## 5 Conclusion

Based on the theory of closed-loop management, our hospital developed a CDSS-based blood transfusion management system integrating workflow control, barcode verification, intelligent warning functions, and structured electronic documentation. The system improved the completeness of transfusion documentation, reduced nurses’ documentation time, and enhanced nurses’ satisfaction with transfusion management. In addition, the CDSS may strengthen standardized monitoring and reporting of transfusion-related adverse reactions through integrated warning and documentation functions, thereby supporting transfusion safety management and improving nurses’ ability to identify and manage transfusion-related events. Overall, the system provides practical support for standardized and information-driven transfusion management in clinical settings.

## 6 Relevance for clinical practice

Hospitals should consider implementing closed-loop blood transfusion management systems supported by clinical decision support technology to strengthen the standardization and traceability of transfusion workflows. Integrating barcode scanning, electronic verification, and intelligent warning functions into nursing information systems can help reduce human errors and support nurses in performing standardized transfusion procedures. In addition, structured electronic transfusion documentation and decision support prompts may assist nurses in identifying and managing transfusion-related adverse reactions more effectively, thereby improving the overall safety and quality of transfusion care.

## Data Availability

The datasets presented in this article are not readily available because Private. Requests to access the datasets should be directed to 3191016@zju.edu.cn.
